# *TFAP2B* Influences the Effect of Dietary Fat on Weight Loss under Energy Restriction

**DOI:** 10.1371/journal.pone.0043212

**Published:** 2012-08-27

**Authors:** Tanja Stocks, Lars Ängquist, Karina Banasik, Marie N. Harder, Moira A. Taylor, Jörg Hager, Peter Arner, Jean-Michel Oppert, J. Alfredo Martinez, Jan Polak, Francis Rousseau, Dominique Langin, Stephan Rössner, Claus Holst, Ian A. MacDonald, Yoichiro Kamatani, Andreas F. H. Pfeiffer, Marie Kunesova, Wim H. M. Saris, Torben Hansen, Oluf Pedersen, Arne Astrup, Thorkild I. A. Sørensen

**Affiliations:** 1 Institute of Preventive Medicine, Copenhagen University Hospital, Frederiksberg, Denmark; 2 Department of Surgical and Perioperative Sciences, Urology and Andrology, Umeå University, Umeå, Sweden; 3 Novo Nordisk Foundation Center for Basic Metabolic Research, Faculty of Health Sciences, University of Copenhagen, Copenhagen, Denmark; 4 School of Biomedical Sciences, Queen's Medical Center, University of Nottingham, Nottingham, United Kingdom; 5 Department of Human Genetics, CEA-Genomics Institute, Évry, France; 6 Department of Medicine, Karolinska Institute, Karolinska University Hospital, Stockholm, Sweden; 7 Department of Nutrition, University Pierre et Marie Curie-Paris 6, Pitie-Salpetriere Hospital (AP-HP), Human Nutrition Research Center Ile-de-France, Paris, France; 8 Department of Physiology and Nutrition, University of Navarra, Pamplona, Spain; 9 Department of Sports Medicine, Center of Preventive Medicine, Third Faculty of Medicine, Charles University, Prague, Czech Republic; 10 IntegraGen SA, Genopole Campus, Évry, France; 11 Obesity Research Laboratory, Institute of Metabolic and Cardiovascular Diseases, Inserm UPS UMR1048, Paul Sabatier University, Toulouse University Hospitals, Toulouse, France; 12 The Obesity Unit, Department of Medicine, Karolinska Institute, Karolinska University Hospital, Huddinge, Sweden; 13 Fondation Jean Dausset, Centre d'Etude du Polymorphisme Humain, Paris, France; 14 Department of Clinical Nutrition, German Institute of Human Nutrition, Nuthetal, Germany; 15 Department of Endocrinology, Diabetes and Nutrition, Charit? Universitaetsmedizin Berlin, Berlin, Germany; 16 Obesity Management Center, Institute of Endocrinology, Prague, Czech Republic; 17 DSM Corporate Scientist, DSM Nutritional Products Ltd, Kaiseraugst, Switzerland; 18 Department of Human Biology, Nutrition and Toxicology Research Institute Maastricht, Maastricht University, Maastricht, The Netherlands; 19 Faculty of Health Sciences, University of Southern Denmark, Odense, Denmark; 20 Institute of Biomedical Science, Faculty of Health Sciences, University of Copenhagen, Copenhagen, Denmark; 21 Faculty of Health Sciences, University of Aarhus, Aarhus, Denmark; 22 Department of Human Nutrition, Faculty of Life Sciences, University of Copenhagen, Frederiksberg, Denmark; Paris Institute of Technology for Life, Food and Environmental Sciences, France

## Abstract

**Background:**

Numerous gene loci are related to single measures of body weight and shape. We investigated if 55 SNPs previously associated with BMI or waist measures, modify the effects of fat intake on weight loss and waist reduction under energy restriction.

**Methods and Findings:**

Randomized controlled trial of 771 obese adults. (Registration: ISRCTN25867281.) One SNP was selected for replication in another weight loss intervention study of 934 obese adults. The original trial was a 10-week 600 kcal/d energy-deficient diet with energy percentage from fat (fat%) in range of 20–25 or 40–45. The replication study used an 8-weeks diet of 880 kcal/d and 20 fat%; change in fat% intake was used for estimation of interaction effects. The main outcomes were intervention weight loss and waist reduction. In the trial, mean change in fat% intake was −12/+4 in the low/high-fat groups. In the replication study, it was −23/−12 among those reducing fat% more/less than the median. *TFAP2B-*rs987237 genotype AA was associated with 1.0 kg (95% CI, 0.4; 1.6) greater weight loss on the low-fat, and GG genotype with 2.6 kg (1.1; 4.1) greater weight loss on the high-fat (interaction p-value; p = 0.00007). The replication study showed a similar (non-significant) interaction pattern. Waist reduction results generally were similar. Study-strengths include (i) the discovery study randomised trial design combined with the replication opportunity (ii) the strict dietary intake control in both studies (iii) the large sample sizes of both studies. Limitations are (i) the low minor allele frequency of the *TFAP2B* polymorphism, making it hard to investigate non-additive genetic effects (ii) the different interventions preventing identical replication-discovery study designs (iii) some missing data for non-completers and dietary intake. No adverse effects/outcomes or side-effects were observed.

**Conclusions:**

Under energy restriction, *TFAP2B* may modify the effect of dietary fat intake on weight loss and waist reduction.

## Introduction

Large genome-wide association studies (GWAS) have identified 32 gene loci in which single nucleotide polymorphisms (SNPs) are robustly, though moderately, related to body mass index (BMI) [1], and fewer variants have been related to proxy measures of abdominal obesity [2]–[4]. All these genetic variants have been related to body size measured at a single time point in adulthood.

Individuals differ greatly in weight loss response to energy restriction [5]–[10]. A large part of the variation is likely explained by variation in adherence to a prescribed diet [9], but differences in physical activity and metabolic response determining energy efficiency may also play a role. Twin studies indicate that genetic predisposition influence weight change response during underfeeding and overfeeding [11]–[13], but few studies have shown effects of various genotypes on weight loss response [14]–[20].

The optimal macronutrient composition of a diet for weight loss has been much debated [21], [22]. We hypothesize that the individual genetic background may influence which diet is the most effective for weight loss. We previously investigated gene-macronutrient interactions in the NUGENOB (Nutrient-Gene Interactions in Human Obesity) study, in which obese men and women were randomized to a 10-weeks hypocaloric diet with low or high fat relative to carbohydrate content. The assigned diets showed similar effects on weight loss [10], but we found that a SNP (rs7903146) of the *TCF7L2* gene associated with diabetes modified the effect of randomized diet on weight loss [16]. However, we found no effect of the obesity-associated SNP of the *FTO* gene on weight loss [15], or, for the time, SNPs in a range of relevant candidate genes in obesity [23] on the diet-weight loss association.

The aim of this study was to examine the effect of SNPs in gene loci with an established association with body size or waist measures, on changes in weight and waist circumference. In NUGENOB we investigated main genetic effects and interactions between SNPs in these gene loci and the randomized low- versus high-fat group, and also for reported changes in energy intake from fat and in total, in relation to weight loss and waist reduction. In order to strengthen or refute findings of strong effects in NUGENOB, we aimed at replication in another weight loss study (Diet, Obesity and Genes, DiOGenes [24]).

## Participants and Methods

### NUGENOB

Information on the NUGENOB trial (http://www.nugenob.com) has been previously described in detail [10]. Briefly, 771 obese men and women recruited to one of eight European centers were randomly assigned to a low- or high-fat diet with corresponding high or low carbohydrate content for ten weeks. Inclusion criteria were a BMI of at least 30 kg/m^2^, and age 20–50 years. Participants were not included if they had experienced a weight change more than 3 kg within three months prior to the study, or if they reported other pre-identified characteristics that could influence the results. Dietary targets were an energy intake of 20–25% from fat (fat%) in the low-fat group, and 40–45 fat% in the high-fat group. Both groups were prescribed a protein intake of 15 energy percentages with no or minimal alcohol consumption, and the goal for energy intake was a daily energy deficit of approximately 600 kcal, calculated by (1.3×resting energy expenditure)-600. Dieticians instructed participants individually about how to reach the assigned diet, and also about how to weigh and record food intake. Weighed food recordings were requested from participants for one weekend day and two weekdays at baseline and at week ten of the intervention, and for one weekday at week two and five. The reported intake was analyzed locally on country-specific food databases. Participants were asked to maintain their usual physical activity during the intervention.

Participants underwent a standardised clinical examination, including anthropometry, at their clinical center shortly before and after the intervention. Participants were instructed to avoid strenuous exercise and abstain from drinking alcohol three days prior to the examination, and to fast overnight and void the bladder before measurements. All variables of diet and anthropometry were checked for unrealistic values and outliers, and such values, two or less for each variable, were excluded.

### Gene loci selection and genotyping

Gene loci included were those that had shown a consistent association with BMI, waist circumference, or waist-hip ratio in GWAS [1]–[4], [25]–[35]. In total 55 SNPs were included in the study. The FTO SNP was genotyped by Taqman allelic discrimination (KBioScience, Herts, UK), with a 96.9% genotyping success rate and a genotyping error rate of 0.27%. Genome-wide genotyping on the Illumina 317 k quad chip carried out at IntegraGen, Evry, France, covered another 23 SNPs in this study, with a 99.1% genotyping success rate. The remaining 31 SNPs were genotyped by KASPar SNP Genotyping (KBioscience, Hoddesdon, UK). Success rate was above 97% and error rate was below 0.5% in 183 replicate samples. We used Fisher's exact test to evaluate Hardy-Weinberg equilibirium (HWE) for all SNPs.

### Statistical analysis

Drop-out rates by genotypes were compared by Pearson chi-square statistics. The baseline BMI of completers and non-completers were compared by two-sample t-test.

We used linear regression to calculate main genotype effects, and gene-diet interactions, in relation to changes in weight and waist. The additive genetic effects and the sum of genotypes, each encoded 0, 1, or 2, equivalent to the number of obesity risk-alleles, were analyzed. Dietary factors investigated were the two randomized groups, and reported change in fat% and energy intake.

Individual change in dietary intake was calculated as the difference between mean intake over three days at baseline, and the intake over five days during the intervention, with equal weight for intake at the first part versus end of intervention. Change in weight and waist circumference corresponded to the difference between levels at baseline and directly after the intervention. All regression models included change in weight or waist as dependent variable, and explanatory variables were genotype, baseline weight or waist, sex, a product term for sex and baseline weight or waist, age, and study center. Analyses of gene-diet interactions additionally included a product term of genotype and the dietary factor of interest and also the dietary factor as main effect. Genetic effects on weight change and waist change are denoted as effects on weight loss and waist reduction.

All statistical tests were two-sided and were performed in Stata 9.2 (StataCorp, LP, College Station, 2007).

### Replication in DiOGenes

The strongest effects observed in NUGENOB were replicated in the DiOGenes study (www.diogenes-eu.org). The trial has been described in detail [24]; briefly, DiOGenes is an intervention study of obese adults undergoing two phases; an 8-weeks weight loss phase on a low-calorie diet, and 6–12 months weight maintenance on one of five diets. Data from the weight loss phase were used in the present study.

Participants were men and women with a BMI between 27 and 45 kg/m^2^, recruited to one of eight European centers. Exclusion criteria were characteristics that could potentially influence the results, such as more than 3 kg weight change within two months prior to the study and medications or certain diseases. Participants of the study recorded their baseline dietary intake during two weekdays and one weekend day. Weighed food recordings were applied with instructions and procedures similar to those in NUGENOB. The 8-weeks weight loss intervention consisted of a low-calorie Modifast diet (Nutrition et Santé). Four items per day were selected from various products, each containing 202–218 kcal. Participants were also allowed a daily intake of 200 g tomatoes, 125 g cucumber, and 50 g lettuce. The low-calorie diet provided approximately 880 kcal/d with an energy-percent from fat of 20%, 54% from carbohydrates, and 26% from protein.

Anthropometric measurements were standardized across the study centers. Height was measured four weeks before the trial. Weight and waist circumference were measured 1–3 weeks before the trial and right after the intervention.

In total 934 participants started the weight loss trial in DiOGenes, and 790 of those had their DNA genome-wide genotyped; missing individuals were due to little or low-quality DNA. Genotyping was performed on the Illumina 660 k quad chip carried out at the Centre National de Génotypage (CNG), Evry, France. Genotyping was successful in 785 (99.4%) individuals. Quality control (duplicates, sex discrepancy, non-European, etc.) reduced the number to 734 (93%) and *TFAP2B* rs987237 was called for all.

Statistical analysis was conducted as described for NUGENOB. Dietary fat in gene-diet interaction analyses was in DiOGenes assessed for change in fat% intake, calculated as the difference between reported baseline intake and the common (approximate) fat proportion of the intervention diet. The inter-individual variation for calculated fat% change was therefore exclusively determined by baseline dietary intake. Median fat% change was used as cut-point for two groups of fat% change, which replaced the randomized fat groups in NUGENOB. Statistical models were identical in all other aspects.

### Ethical considerations

The NUGENOB and DiOGenes trials have obtained written informed consent from all study participants, and ethical approval from the ethical committee at each study center. The names of the local ethics committees were: (NuGenOb trial) Research Ethics Committee of the University of Navarra, Spain; The Danish Research Ethics Committee System, Denmark; Medical Ethics Committee of the Maastricht University, Netherlands; Ethics Committee of the Hôtel-Dieu Hospital, France; University of Nottingham Medical School Ethics Committee, United Kingdom; The Swedish National Council on Medical Ethics, Sweden; Ethics Commission of the Medical School of Toulouse, France. (DiOGenes trial) Research Ethic Committee of the University of Navarra, Spain; The Danish Research Ethics Committee System, Denmark; Medical Ethics Committee of the Maastricht University, Netherlands; Ethics Committee of the University of Potsdam, Germany; Bedfordshire Local Research Ethics Committee, United Kingdom;

Research Ethics Committee of the University of Crete, Greece; Ethical Committee to the National Transport Multiprofile Hospital in Sofia, Bulgaria; Ethical Committee of the Faculty Hospital, Institute of Endocrinology, Prague, Czech Republic.

## Results

### NUGENOB

**Figure 1:**
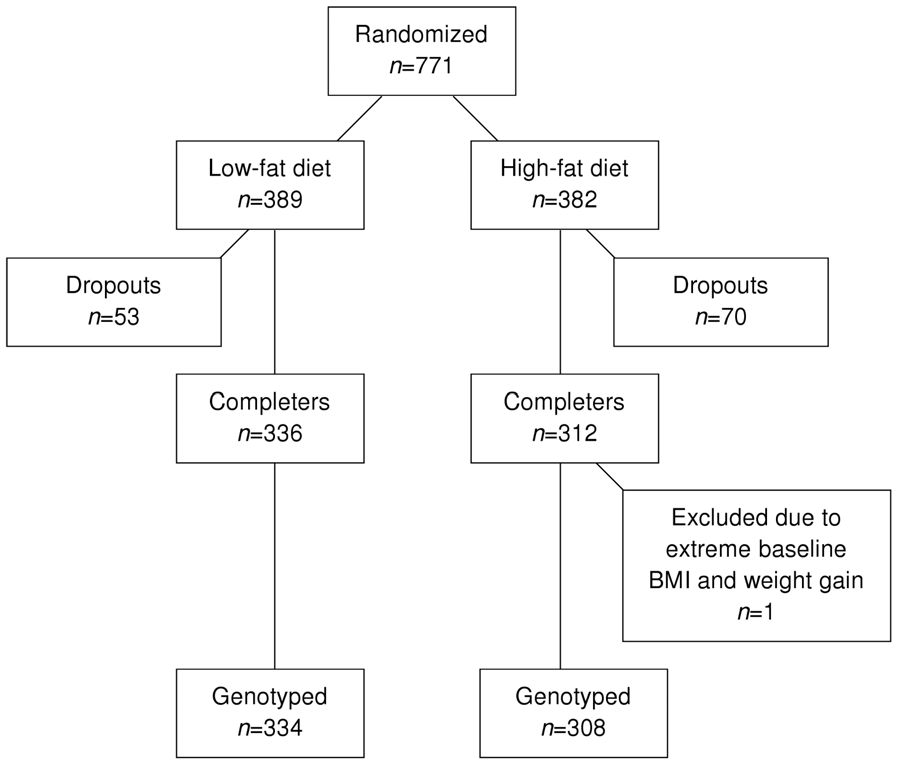
Participant flow in NUGENOB.

Out of 771 participants at start of the intervention, 648 (84%) completed it (**Figure 1**). Completers and non-completers did not differ significantly in baseline BMI (*P* = 0.8). One weight gainer with an extreme baseline BMI of 66 kg/m^2^ and five participants with missing genotype data were excluded. For each of the 55 variants included in the study, genotype data were available in 617–642 participants (95–99% of completers). Genotype distributions were not related to drop-out rates except for *ETV5* and *FAIM2*, which had more completers among homozygotes than among non-carriers of the obesity risk-allele (*P* = 0.01 and 0.02 respectively), and for *PTBP2*, which showed the opposite pattern (*P* = 0.03). Of completers with genotype data, 580 (90% of 642) participants had dietary intake data available, and they were included in gene-diet interaction analyses that included reported dietary intake.

Participants had a mean baseline BMI of 35.5 kg/m^2^, and mean weight was 96.8 kg in women and 110.6 kg in men (**Table 1**). Energy intake reduced by on average 615 kcal/d in the study group. This was achieved by a reduction in absolute intake of macronutrients, with a greater decrease in fat intake in the low-fat group, and a greater decrease of carbohydrates in the high-fat group. On average, women lost 6.5 kg weight and 5.9 cm waist and men lost 7.9 kg weight and 7.7 cm waist during the intervention.

**Table 1:** Descriptive information with respect to the NUGENOB study.

Variable	Women (*n* = 481)		Men (*n* = 161)		All (*n* = 642)	
	Baseline	Change*	Baseline	Change*	Baseline	Change*
Age, years	36.5 (7.9)		38.7 (7.6)		37.1 (7.9)	
Weight, kg	96.8 (14.5)	−6.5 (3.2)	110.6 (16.3)	−7.9 (3.8)	100.3 (16.1)	−6.8 (3.4)
BMI, kg/m^2^	35.8 (4.8)	−2.4 (1.2)	34.7 (4.5)	−2.5 (1.2)	35.5 (4.7)	−2.4 (1.2)
Waist circumference, cm†	103.2 (12.0)	−5.9 (4.5)	113.9 (11.4)	−7.7 (4.3)	105.9 (12.7)	−6.3 (4.5)

	Low-fat diet (*n* = 298)		High-fat diet (*n* = 282)		All (*n* = 580)	
	Baseline	Change‡	Baseline	Change‡	Baseline	Change‡
Energy intake, kcal/d	2201 (691)	−652 (632)	2194 (625)	−576 (534)	2197 (659)	−615 (587)
Fat, E%§	36.7 (7.6)	−11.6 (8.2)	37.0 (7.8)	3.9 (9.0)	36.9 (7.7)	−4.1 (11.6)
Fat, g	90.9 (37.3)	−47.4 (34.8)	91.2 (35.7)	−17.5 (33.2)	91.1 (36.5)	−32.9 (37.2)
Carbohydrates, E%§	45.2 (8.6)	11.5 (8.9)	44.8 (8.7)	−2.9 (9.5)	45.0 (8.6)	4.5 (11.7)
Carbohydrates, g	246.7 (86.4)	−27.2 (85.3)	245.0 (80.2)	−75.8 (76.7)	245.9 (83.4)	−50.9 (84.7)
Protein, E%§	16.3 (3.4)	1.6 (3.6)	16.3 (3.2)	0.7 (3.6)	16.3 (3.3)	1.2 (3.6)
Protein, g	87.7 (27.6)	−18.7 (26.0)	88.4 (27.6)	−19.7 (23.9)	88.1 (27.6)	−19.2 (25.0)

Mean (SD) baseline level and change of body size by sex in 642 completers in NUGENOB with genotype data available, and dietary intake by randomized group in 580 completers with genotype data and dietary intake data available.

E%; percent of total energy intake.

* Difference between baseline and after the intervention.

† Data were missing in 14 participants.

‡ Difference between reported intake at baseline and during the intervention.

§ Alcohol contributed with on average 1.8 E% (6.3 g) at baseline and the proportion reduced during the intervention.

The distribution of genotypes, allele frequency, and outcome of HWE test for the 55 gene loci are reported in **Table S1**. All minor allele frequencies were larger than 0.05, and HWE tests were acceptable with only one SNP (*PFKP* rs6602024) showing a *P*-value below 0.01.

**Figure 2:**
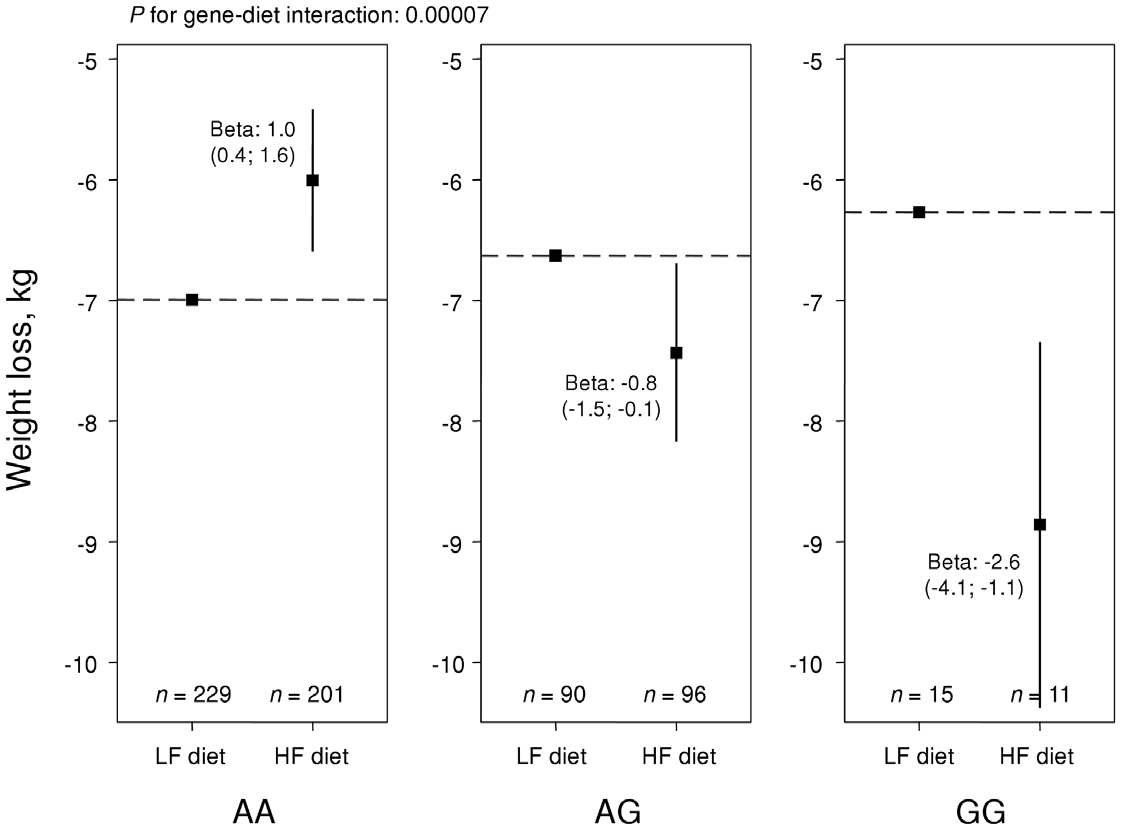
Weight loss over *TFAP2B* rs987237 genotypes in NUGENOB. Effect of randomized fat group (low-fat, LF, and high-fat, HF) on weight loss in NUGENOB by *TFAP2B* rs987237. The y-axis displays the mean weight loss in each group. *P*-values for interaction, and effect estimates, were derived from linear regression, based on the assumption of an additive genetic model.

**Figure 3:**
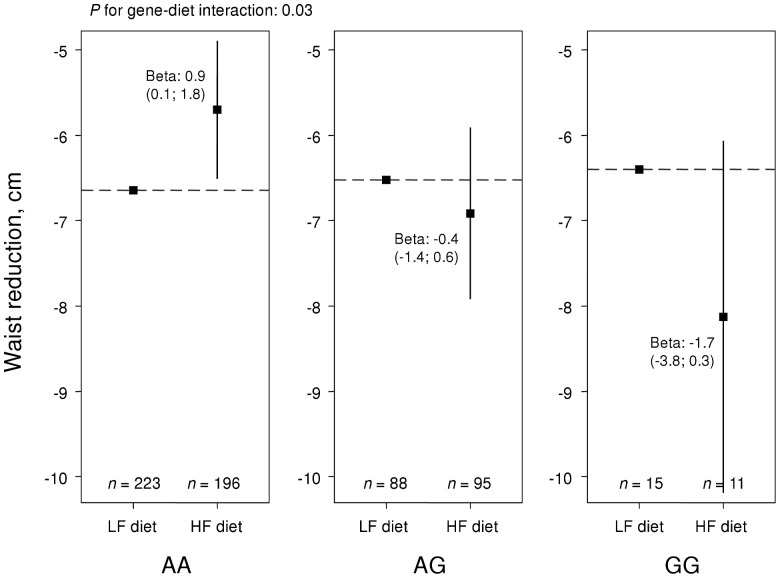
Waist reduction over *TFAP2B* rs987237 genotypes in NUGENOB. Effect of randomized fat group (low-fat, LF, and high-fat, HF) on waist reduction in NUGENOB by *TFAP2B* rs987237. The y-axis displays the mean waist reduction in each group. *P*-values for interaction, and effect estimates, were derived from linear regression, based on the assumption of an additive genetic model.

**Table 2** shows *P*-values for main effects and interactions with diet for 17 gene loci that reached nominal *P*-values below 0.05 for an association with weight loss or waist reduction. The most striking results were found for *TFAP2B* rs987237. This SNP modified the effect on weight loss by fat group with a nominal *P*-value for interaction of 0.00007, which also reached significance after correction for multiple testing of 55 SNPs (0.05/55 = 0.0009). In an additive gene-diet interaction model, homozygotes for the A allele lost 1.0 kg (95% confidence interval, CI, 0.4; 1.6) more weight on the low-fat than on the high-fat diet, whereas homozygotes for the G allele, i.e. the obesity risk-allele, lost 2.6 kg (95% CI, 1.1; 4.1) more weight on the high-fat diet (**Figure 2**). A similar but weaker pattern was observed for waist reduction (*P* for interaction = 0.03; **Figure 3**). *TFAP2B* was also directly related to weight loss (*P* = 0.04) with a 0.5 kg (95% CI, 0.0; 0.9) greater weight loss per G allele.

**Table 2:** Regression results with respect to the NUGENOB study.

Gene†	Phenotypechange	All participants (*n* = 617–642)		Participants with dietary data(*n* = 559–580)	
		Genotype effect‡	Gene×low-/high-fat diet§	Gene×change∥ in fat% intake§	Gene×change∥ in energy intake§
*CTNNBL1*	Weight	0.4	0.1	0.7	0.8
	Waist	0.5	**0.0003**	0.05	0.2
*FANCL*	Weight	0.2	0.6	0.8	*0.03*
	Waist	0.5	0.5	0.9	0.9
*GPRC5B*	Weight	0.2	0.1	*0.046*	0.2
	Waist	0.4	0.5	0.5	0.6
*LRRN6C*	Weight	0.3	*0.01*	0.1	0.5
	Waist	0.9	*0.02*	0.4	0.9
*MAF*	Weight	0.7	*0.04*	0.1	0.09
	Waist	0.4	0.1	0.2	0.05
*MAP2K5*	Weight	0.9	1.0	0.3	0.9
	Waist	0.4	0.7	0.9	*0.03*
*MTIF3*	Weight	0.1	0.4	0.3	0.6
	Waist	*0.04*	0.8	0.5	0.7
*NPC1*	Weight	0.7	*0.04*	0.2	1.0
	Waist	0.1	**0.008**	0.9	0.6
*SLC39A8*	Weight	0.7	0.4	0.8	0.9
	Waist	0.3	0.2	*0.03*	0.7
*ZNF608*	Weight	0.06	0.6	0.9	0.1
	Waist	*0.01*	0.5	0.6	0.7
*MC4R*	Weight	0.09	0.3	0.4	*0.049*
	Waist	0.5	0.9	0.9	0.2
*TFAP2B*	Weight	*0.04*	**0.00007**	*0.03*	0.8
	Waist	0.1	*0.03*	0.2	0.5
*ADAMTS9*	Weight	0.9	0.2	0.2	0.6
	Waist	0.2	*0.04*	0.1	1.0
*DNM3-PIGC*	Weight	0.8	0.6	0.2	*0.02*
	Waist	0.3	0.3	0.8	0.2
*LY86*	Weight	*0.02*	0.4	0.6	0.2
	Waist	0.1	0.7	0.7	0.3
*RSPO3*	Weight	0.3	0.1	0.5	*0.01*
	Waist	0.3	*0.045*	0.2	0.3
*VEGFA*	Weight	**0.001**	0.2	0.4	0.9
	Waist	*0.03*	0.6	0.7	0.9

*P*-value for main genotype effect and interaction between genotype and diet in relation to weight loss and waist reduction for 17 gene loci with a *P*-value<0.05* for one or more associations.

* *P*-values 0.01 to 0.05 are highlighted in italics and *P*-values<0.01 are highlighted in bold.

† See SNP information in Table S1.

‡ *P*-value for the additive effect of genotype adjusted for baseline weight or waist (weight/waist), sex, baseline weight/waist×sex, age, and center.

§ *P*-value for the product term of genotype and the dietary factor of interest, adjusted for baseline weight/waist, sex, baseline weight/waist×sex, age, center, genotype, and the dietary factor of interest.

∥ Difference between reported intake at baseline and during the intervention.

Three other gene loci showed *P*-values between 0.0003 and <0.01 for a main effect or interaction with diet, in relation to weight loss or waist reduction (**Table S2**). These were main effects on weight loss by *VEGFA*, and a gene-fat group interaction in relation to waist reduction for *CTNNBL1* and *NPC1*.

The gene score (sum of all the risk alleles over genotypes) showed no main effect or interaction with diet in relation to outcomes (*P*≥0.09).

### Replication of *TFAP2B*-fat interaction analyses in DiOGenes

Out of 803 (86%) completers of the intervention, data on *TFAP2B* were available in 639 participants, of which 590 participants had reported their dietary intake before the intervention. Completers did not differ significantly from non-completers with respect to BMI (P = 0.7) or *TFAP2B* genotype distribution (P = 0.4).

Characteristics of DiOGenes participants are shown in **Table 3**. Whereas the range for change in fat% intake in NUGENOB was broad and largely related to randomized group, fat intake among DiOGenes participants reduced much both in terms of absolute intake (mean: −74.1 g/d, range: −251.2 to −0.1 g) and fat% (mean: −17.3, range −43.7 to 1.7). The mean energy intake reduction of 1351 kcal/d in DiOGenes was more than double the reduction in NUGENOB, and weight loss and waist reduction was greater.

**Table 3:** Descriptive information with respect to the DiOGenes study.

Variable	Women (*n* = 415)		Men (*n* = 224)		All (*n* = 639)	
	Baseline	Change*	Baseline	Change*	Baseline	Change*
Age, years	41.0 (6.3)		43.0 (5.5)		42.0 (6.1)	
Weight, kg	94.9 (15.4)	−9.9 (3.0)	109.5 (17.3)	−12.8 (4.1)	100.0 (17.5)	−10.9 (3.7)
BMI, kg/m^2^	34.5 (4.9)	−3.7 (1.0)	34.4 (4.6)	−4.0 (1.2)	34.5 (4.8)	−3.8 (1.1)
Waist circumference, cm†	104.3 (11.8)	−9.1 (4.7)	114.6 (12.4)	−11.1 (4.3)	107.9 (13.0)	−9.8 (4.7)

	>16.7 fat% decrease(*n* = 295)		<16.7 fat% decrease‡(*n* = 295)		All (*n* = 590)	
	Baseline	Change§	Baseline	Change§	Baseline	Change§
Energy intake, kcal/d	2307 (774)	−1427 (774)	2154 (778)	−1274 (777)	2231 (779)	−1351 (779)
Fat, E%∥	43.5 (5.1)	−23.0 (5.1)	32.0 (3.8)	−11.6 (3.8)	37.8 (7.3)	−17.3 (7.3)
Fat, g	111.1 (40.5)	−91.1 (40.5)	77.4 (31.3)	−57.4 (31.3)	94.1 (39.8)	−74.1 (39.8)
Carbohydrates, E%∥	38.0 (6.2)	15.7 (6.2)	47.6 (7.2)	6.0 (7.2)	42.8 (8.3)	10.8 (8.3)
Carbohydrates, g	218.1 (77.6)	−100.1(77.6)	257.2(100.3)	−139.2(100.3)	237.5(91.7)	−119.5(91.7)
Protein, E%∥	17.1 (4.0)	8.8 (4.0)	17.0 (4.5)	8.9 (4.5)	17.1 (4.3)	8.8 (4.3)
Protein, g	96.5 (34.5)	−39.5 (34.5)	88.3 (30.3)	−31.3 (30.3)	92.4 (32.7)	−35.4 (32.7)

Mean (SD) baseline level and change of body size by sex in 639 completers in DiOGenes in whom *TFAP2B* was successfully genotyped, and dietary intake in groups split by median change in fat% intake in 590 completers with *TFAP2B* data and dietary intake data available.

E%; percent of total energy intake.

* Difference between baseline and after the intervention.

† Data were missing in 41 participants.

‡ One participant reported an increased (1.7 units) fat% intake.

§ Difference between reported intake at baseline and the standardised low-calorie diet of 880 kcal/day, including macronutrients: fat, 20 g, 20 E%; carbohydrates, 118 g, 54 E%; and protein, 57 g, 26 E%.

∥ Alcohol contributed with on average 2.3 E% (7.9 g) at baseline and no alcohol was allowed during the intervention.

**Figure 4:**
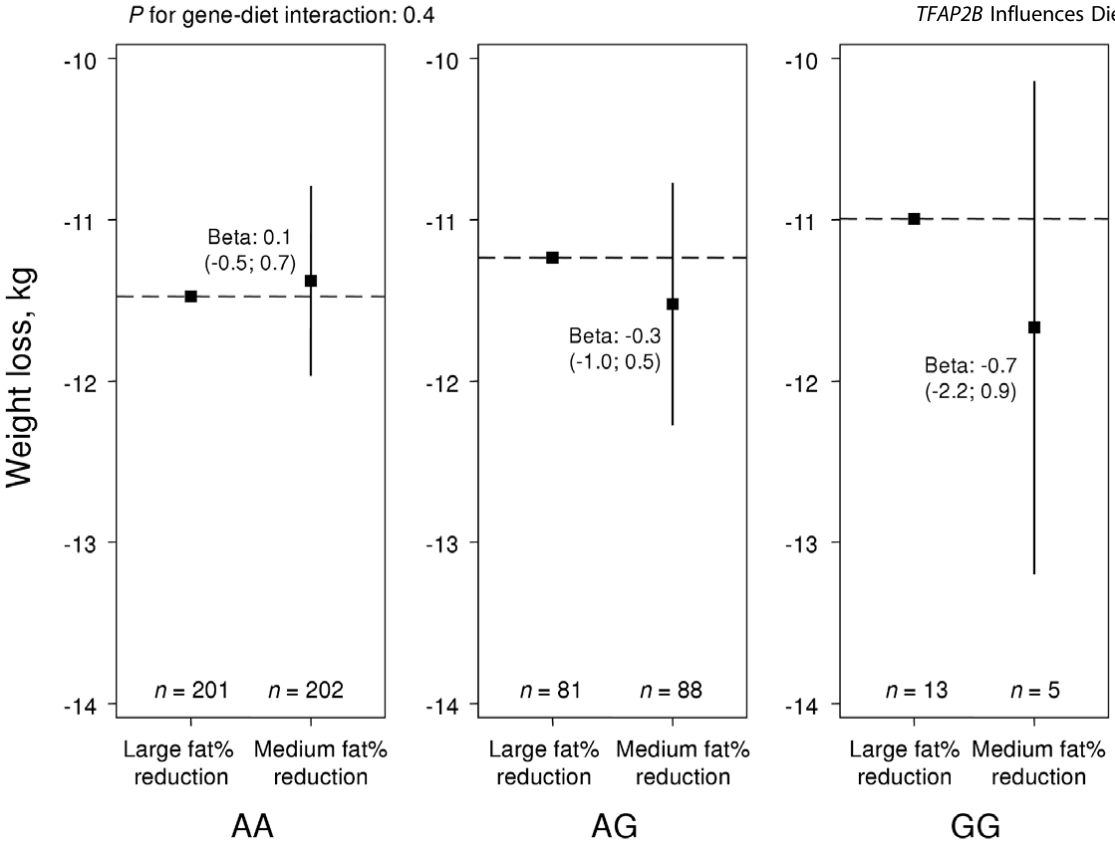
Weight loss over *TFAP2B* rs987237 genotypes in DiOGenes. Effect of fat% reduction on weight loss in DiOGenes by *TFAP2B* rs987237. The two groups of fat% reduction were divided by the median reduction of 16.7 energy percent. The y-axis displays the mean weight loss in each group. *P*-values for interaction, and effect estimates, were derived from linear regression, based on the assumption of an additive genetic model.

**Figure 5:**
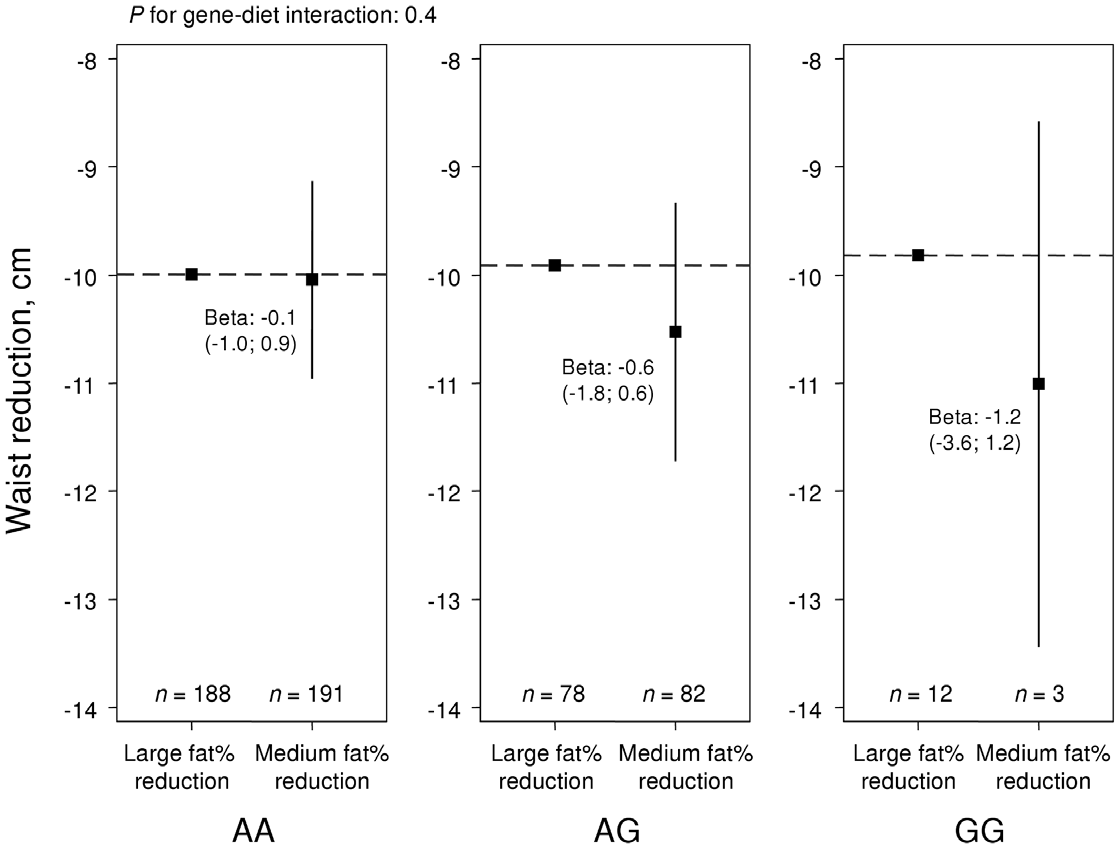
Waist reduction over *TFAP2B* rs987237 genotypes in DiOGenes. Effect of fat% reduction on waist reduction in DiOGenes by *TFAP2B* rs987237. The two groups of fat% reduction were divided by the median reduction of 16.7 energy percent. The y-axis displays the mean waist reduction in each group. *P*-values for interaction, and effect estimates, were derived from linear regression, based on the assumption of an additive genetic model.

Analysis of *TFAP2B* combined with a fat% reduction above or lower than the median of 16.7 fat% showed similar patterns for associations with weight loss (**Figure 4**) and waist reduction (**Figure 5**) to those in NUGENOB, but statistical evidence for interaction was weak (*P* = 0.4). There were no main effects of *TFAP2B* on weight loss (*P* = 0.8) or waist reduction (*P* = 0.6).

Additional robustness analyses in NUGENOB and DiOGenes of the *TFAP2B*-dietary fat interaction are described in supporting material **Text S1** and displayed in **Table S3 and S4**.

## Discussion

In this study of weight loss in obese men and women on a 10-weeks energy restricted diet low or high in fat to carbohydrate content, we investigated main effects and gene-diet interactions of 55 gene loci with an established association with BMI or waist measures at genome-wide significant levels. We found that the *TFAP2B* gene locus rs987237 clearly modified the effect of a high versus low-fat diet on weight loss. Whilst non-carriers of the obesity risk-allele lost more weight on the low-fat diet, the opposite was shown among homozygotes of the obesity risk-allele. These results showed a similar, though non-significant, pattern in another weight loss study of a low-calorie diet for eight weeks when comparing participants with a small versus large reduction of fat% intake. The similar pattern of results from these different studies indicates that under energy restriction, *TFAP2B* may influence the effect of dietary fat on weight loss.

Several factors may underlie the much weaker associations observed in the replicated analysis. Analyses in NUGENOB were performed in line with the original design; two randomized groups of low or high fat% intake were compared. Besides the control of unknown confounders owing to randomization, the two groups in NUGENOB were also more diverse in their changed fat% intake than were the constructed groups in DiOGenes with different decreases in dietary fat% intake. Moreover, we lacked information on the actual individual dietary intake during the intervention in DiOGenes, so dietary change calculations assumed complete adherence to the prescribed diet and this assumption is likely not to be entirely met. Given these limitations to enable replication of the findings in NUGENOB, the similar pattern observed in DiOGenes is quite noteworthy. Many of the same limitations in DiOGenes confer also to NUGENOB for investigation of change in fat% and energy intake. Misclassification of self-reported dietary intake, small variation in exposure or small differences between compared groups, and confounding issues may have obscured the finding of further strong gene-diet interactions in NUGENOB, and a clearer replication of *TFAP2B*-dietary fat interaction in DiOGenes.

Other limitations of the study are the incomplete data due to drop-outs of study participants, and incomplete genotyping and dietary intake reports. Around 15% of study participants did not complete the trials; however, completers and non-completers did neither differ in baseline BMI nor in overall genotype distribution. Dietary intake data were incomplete in both studies, but were based on weighed food records over several days that, despite its weaknesses owing to self-report, is frequently used as gold standard method for assessment of dietary intake [36].

The major strength of the study is the relatively large sample size of two well-controlled intervention studies of similar participant characteristics, allowing replication of analyses for highly significant findings. Replication is crucial to avoid spurious chance findings emerging from multiple testing. Although analyses in DiOGenes did not replicate the strong findings in NUGENOB, the patterns of results were consistent.

The *TFAP2B* gene has been associated with type 2 diabetes [37], and was recently found to be related to BMI [1] and waist circumference [4]. The *TFAP2B* gene encodes for a transcriptional factor-activating enhancer-binding protein-2β (AP-2β) and is preferentially expressed in adipose tissue [37]. Overexpression of *TFAP2B* leads to increased glucose uptake and thereby triglyceride accumulation and insulin resistance in adipocytes [38]. The *TFAP2B* rs987237 variant investigated in this study, located in intron 3, may not be the causal variant. It may, however, be a marker of the enhancer activity, since it is in complete linkage disequilibrium (r^2^ = 1) with an intronic enhancer variant that influences *TFAP2B* expression in adipose tissue [39]. Yet, the mechanisms whereby *TFAP2B* could modify the effect of dietary fat intake on weight loss are unclear. It is also unclear whether *TFAP2B* indeed interacts with dietary fat, as focused on in our paper, or rather with carbohydrates, or the fat-carbohydrate ratio. Speculatively, individuals of different *TFAP2B* genotype and gene expression metabolise fat or other macronutrients differently, and thus, respond differently to dietary change of macronutrient composition.

In conclusion, this study of obese men and women showed a clear interaction between *TFAP2B* and a diet low or high in fat, on weight loss under energy restriction. A similar, but nonsignificant, pattern for interaction between *TFAP2B* and dietary fat intake was shown in another weight loss study of similar participant characteristics. Results of our study strongly encourage further examination of the role of *TFAP2B* and macronutrients in weight loss.

## Supporting Information

Figure S1 **Weight loss over** ***TFAP2B*** **rs987237 genotypes in NUGENOB based on general genetic model.** Effect of randomized fat group (low-fat, LF, and high-fat, HF) on weight loss in NUGENOB by *TFAP2B* rs987237. The y-axis displays the mean weight loss in each group. *P*-values for interaction, and effect estimates, were derived from linear regression, based on the assumption of a general genetic model (SNP coded as a categorical variable).
(TIF)

Figure S2 **Waist reduction over** ***TFAP2B*** **rs987237 genotypes in NUGENOB based on general genetic model.** Effect of randomized fat group (low-fat, LF, and high-fat, HF) on waist reduction in NUGENOB by *TFAP2B* rs987237. The y-axis displays the mean waist reduction in each group. *P*-values for interaction, and effect estimates, were derived from linear regression, based on the assumption of a general genetic model (SNP coded as a categorical variable).
(TIF)

Table S1 **Genotype information of the 55 gene loci included in the study.**
(PDF)

Table S2 **Genes with interesting findings (** ***P*** **<0.01).** Gene and gene-diet interaction effects with *P*<0.01 in relation to change in weight or waist.
(PDF)

Table S3 **Sensitivity analysis for weight loss over** ***TFAP2B*** **rs987237 genotypes in NUGENOB.** *P*-value for interaction between *TFAP2B* and fat group, and beta (95% confidence interval) for fat group by *TFAP2B* variant, in relation to weight loss, in analyses varying in fat group definition and adjustments, in NUGENOB.
(PDF)

Table S4 **Sensitivity analysis for weight loss over** ***TFAP2B*** **rs987237 genotypes in DiOGenes.** *P*-value for interaction between *TFAP2B* and fat group, and beta (95% confidence interval) for fat group by TFAP2B variant, in relation to weight loss, in analyses varying in fat group definition and adjustments, in DiOGenes.
(PDF)

Text S1 **Outline of sensitivity analysis for weight loss over** ***TFAP2B*** **rs987237 genotypes.** Includes descriptions of both additional sensitivity analyses for the *TFAP2B*-dietary fat interaction (results in **Table S3 and S4**) as well as of analyses based on the general genetic model (SNP coded as a categorical variable, results displayed in **Figure S1 and S2**).
(PDF)
