# Supplementary figures and images for: TFAP2B Influences the Effect of Dietary Fat on Weight Loss under Energy Restriction
Source: PLoS One. 2012 Aug 27;7(8):e43212. doi: 10.1371/journal.pone.0043212 (PMC3428346; doi:10.1371/journal.pone.0043212)

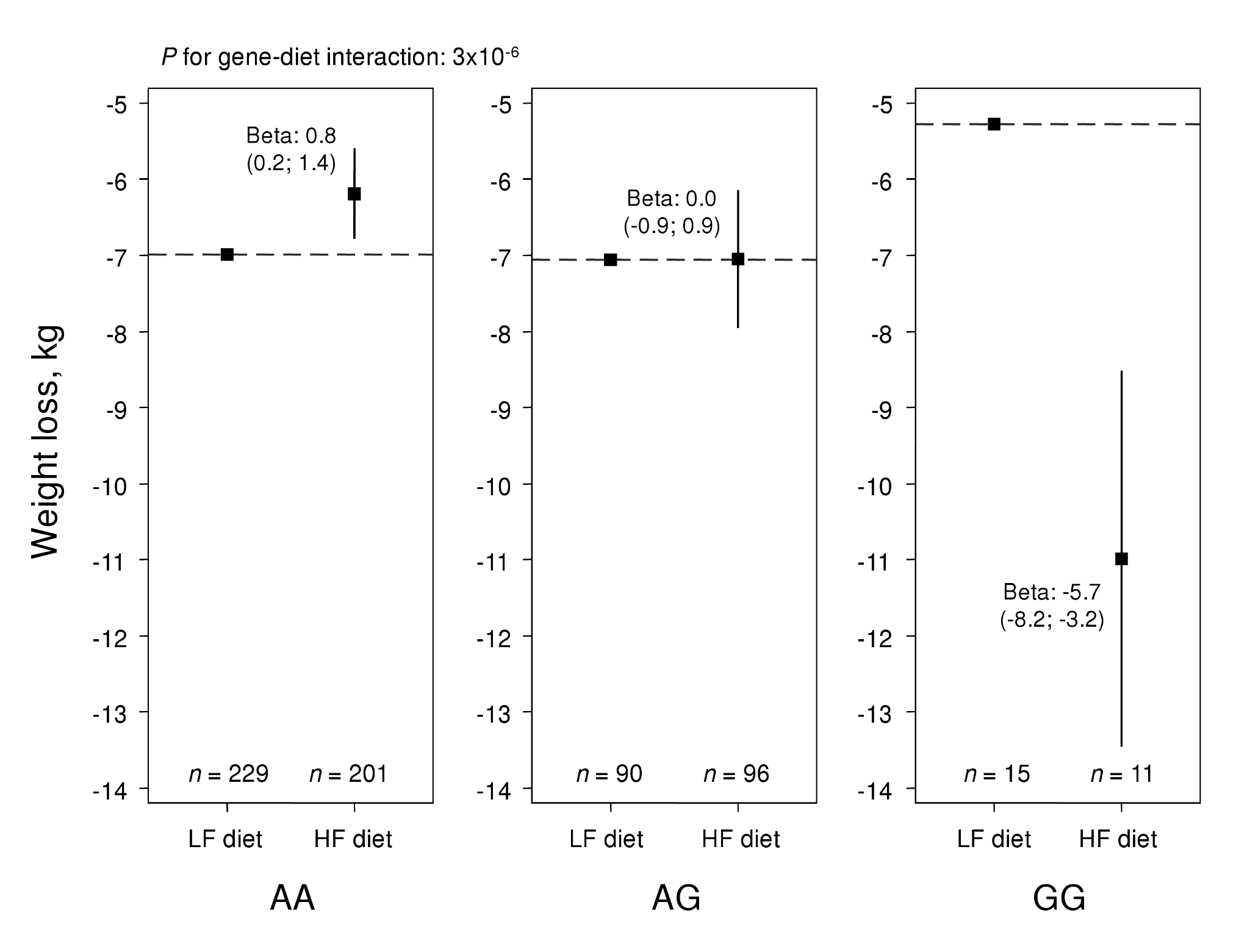

Supplement: Figure S1 — Weight loss over TFAP2B rs987237 genotypes in NUGENOB based on general genetic model. Effect of randomized fat group (low-fat, LF, and high-fat, HF) on weight loss in NUGENOB by TFAP2B rs987237. The y-axis displays the mean weight loss in each group. P-values for interaction, and effect estimates, were derived from linear regression, based on the assumption of a general genetic model (SNP coded as a categorical variable). (TIF) [file pone.0043212.s001.tif]

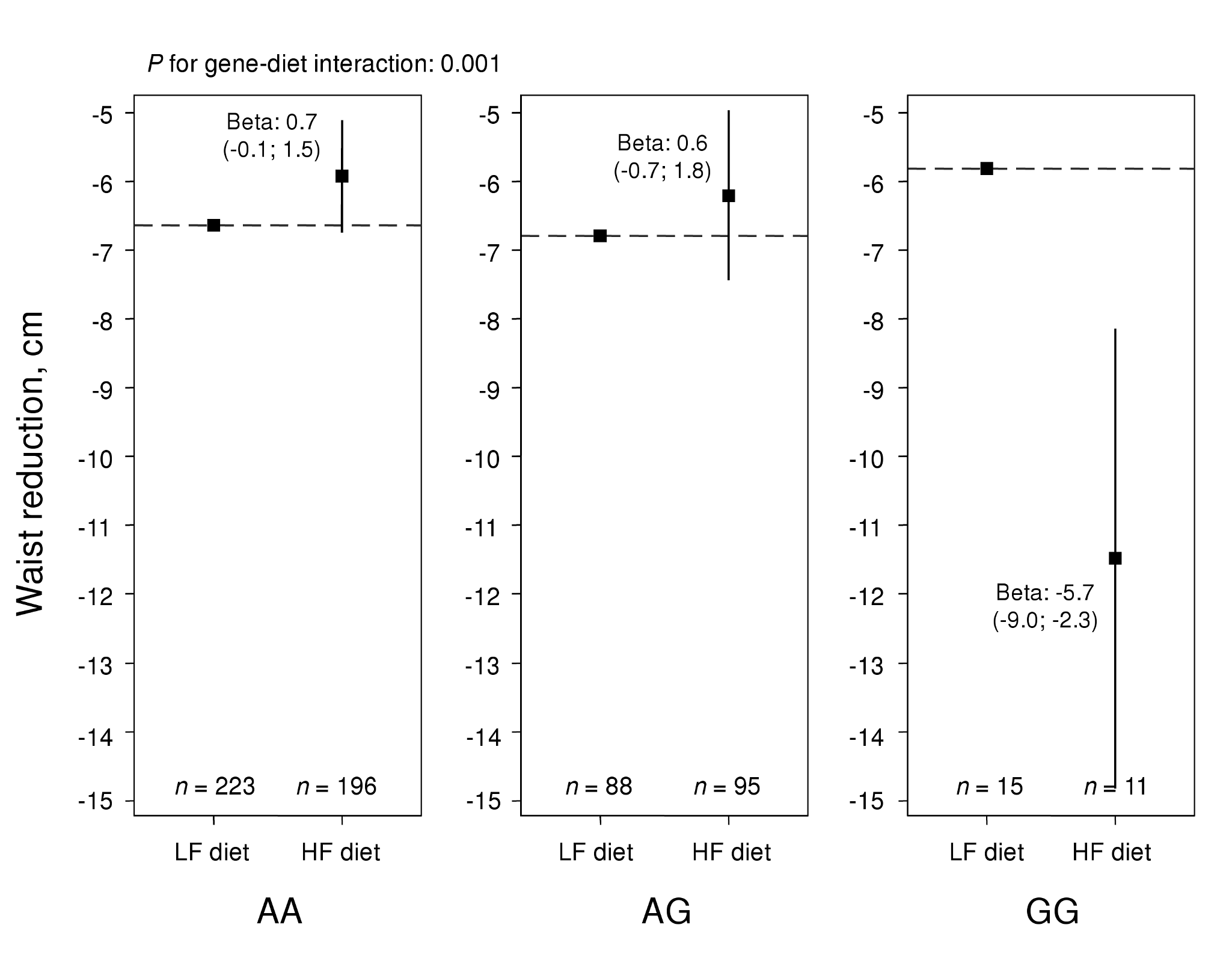

Supplement: Figure S2 — Waist reduction over TFAP2B rs987237 genotypes in NUGENOB based on general genetic model. Effect of randomized fat group (low-fat, LF, and high-fat, HF) on waist reduction in NUGENOB by TFAP2B rs987237. The y-axis displays the mean waist reduction in each group. P-values for interaction, and effect estimates, were derived from linear regression, based on the assumption of a general genetic model (SNP coded as a categorical variable). (TIF) [file pone.0043212.s002.tif]
