# Supplementary material for: TFAP2B Influences the Effect of Dietary Fat on Weight Loss under Energy Restriction
Source: PLoS One. 2012 Aug 27;7(8):e43212. doi: 10.1371/journal.pone.0043212 (PMC3428346; doi:10.1371/journal.pone.0043212)
Supplement: Table S1 — Genotype information of the 55 gene loci included in the study. (PDF) [file pone.0043212.s003.pdf]

Table S1. Genotype information of the 55 gene loci included in the study

Gene	SNP	Reference/-s, (first author, year)[+]	Trait	Risk-allele	Genotype*			Hardy-Weinberg	
					NC	HEZ	HOZ	MAF	*P*-value†
BDNF	rs4923461	Thorleifsson, 2009 [26]; Croteau-Chonka, 2011 [27]; Speliotes, 2010 [1]	BMI	A	32	210	385	0.22	0.6
CADM2	rs13078807	Speliotes, 2010[1]	BMI	G	392	202	24	0.20	0.7
CTNNBL1	rs6013029	Liu, 2008 [28]	BMI	T	555	77	3	0.07	0.9
ETV5	rs9816226	Speliotes, 2010 [1]	BMI	T	9	192	423	0.17	0.01
FANCL	rs887912	Speliotes, 2010 [1]	BMI	T	308	264	63	0.31	0.6
FLJ35779	rs2112347	Speliotes, 2010 [1]	BMI	T	71	289	271	0.34	0.6
GNPDA2	rs10938397	Speliotes, 2010 [1]	BMI	G	198	302	129	0.45	0.5
GPRC5B	rs12444979	Speliotes, 2010 [1]	BMI	C	6	131	492	0.12	0.4
KCTD15	rs29941	Speliotes, 2010 [1]	BMI	G	53	280	288	0.31	0.2
LRP1B	rs2890652	Speliotes, 2010 [1]	BMI	C	448	167	16	0.16	0.9
LRRN6C	rs10968576	Speliotes, 2010 [1]	BMI	G	292	271	62	0.32	0.9
MAF	rs1424233	Meyre, 2009 [32]	BMI	A	170	299	151	0.48	0.4
MAP2K5	rs2241423	Speliotes, 2010 [1]	BMI	G	21	224	381	0.21	0.08
MTCH2	rs10838738	Willer, 2008 [31]; Speliotes, 2010 [1]	BMI	G	250	289	85	0.37	0.9
MTIF3	rs4771122	Speliotes, 2010 [1]	BMI	G	359	230	40	0.25	0.7
NEGR1	rs2568958	Thorleifsson, 2009 [26]; Speliotes, 2010 [1]	BMI	A	94	280	253	0.37	0.3
NPC1	rs1805081	Meyre, 2009 [32]	BMI	A	89	292	244	0.38	0.9
NUDT3	rs206936	Speliotes, 2010 [1]	BMI	G	386	214	32	0.22	0.7
PCSK1	rs6235	Benzinou, 2008 [25]	BMI	C	368	217	45	0.24	0.1
PFKP	rs6602024	Scuteri, 2007 [34]	BMI	A	511	110	15	0.11	0.003
PRKD1	rs11847697	Speliotes, 2010 [1]	BMI	T	569	66	0	0.14	0.4
PRL	rs4712652	Meyre, 2009 [32]; Nilsson, 2011 [35]	BMI	A	130	286	214	0.43	0.06
PTBP2	rs1555543	Speliotes, 2010 [1]	BMI	C	116	318	199	0.43	0.6
PTER	rs10508503	Meyre, 2009 [32]	BMI	C	5	93	526	0.08	0.7
QPCTL	rs2287019	Speliotes, 2010 [1]	BMI	C	24	189	411	0.19	0.7
RBJ	rs713586	Speliotes, 2010 [1]	BMI	C	172	300	158	0.49	0.2
RPL27A	rs4929949	Speliotes, 2010 [1]	BMI	C	157	321	157	0.50	0.8
SEC16B	rs10913469	Thorleifsson, 2009 [26]; Speliotes, 2010 [1]	BMI	C	430	174	22	0.17	0.4
SH2B1	rs7498665	Thorleifsson, 2009 [26]; Willer, 2008 [31]; Speliotes, 2010 [1]	BMI	G	220	271	126	.42	0.01
SLC39A8	rs13107325	Speliotes, 2010 [1]	BMI	T	535	88	4	.08	0.9
TMEM160	rs3810291	Speliotes, 2010 [1]	BMI	A	60	278	282	.32	0.5
TMEM18	rs2867125	Speliotes, 2010 [1]; Thorleifsson, 2009 [26]	BMI	G	15	158	453	.15	0.8
TNNI3K	rs1514175	Speliotes, 2010 [1]	BMI	A	198	309	121	.44	1.0

ZNF608	rs4836133	Speliotes, 2010 [1]	BMI	A	190	284	155	.47	0.02
FAIM2	rs7138803	Speliotes, 2010 [1]; Thorleifsson, 2009 [26]	BMI, WC	A	244	295	86	.37	0.8
FTO	rs9939609	Frayling, 2007 [29]; Speliotes, 2010 [1]; Li, 2010 [30]	BMI, WC	A	205	311	102	.42	0.4
MC4R	rs12970134	Thorleifsson, 2009 [26] ; Chambers, 2008 [33]; Speliotes, 2010 [1]	BMI, WC	A	298	272	57	.31	0.7
NRXN3	rs10146997	Heard-Costa, 2009 [2]; Speliotes, 2010 [1]	BMI, WC	G	390	221	31	.22	1.0
TFAP2B‡	rs987237	Speliotes, 2010 [1]; Lindgren, 2009 [4]	BMI, WC	G	430	186	26	.19	0.3
MSRA	rs545854	Lindgren, 2009 [4]	WC	G	462	161	14	.15	1.0
ADAMTS9	rs6795735	Heid, 2010 [3]	WHR	C	126	313	188	.45	0.8
CPEB4	rs6861681	Heid, 2010 [3]	WHR	A	320	261	53	.29	1.0
DNM3-PIGC	rs1011731	Heid, 2010 [3]	WHR	G	235	283	102	.39	0.3
GRB14	rs10195252	Heid, 2010 [3]	WHR	T	110	283	230	.40	0.2
HOXC13	rs1443512	Heid, 2010 [3]	WHR	A	359	238	29	.24	0.2
ITPR2-SSPN	rs718314	Heid, 2010 [3]	WHR	G	350	223	46	.25	0.2
LYPLAL1	rs2605100	Lindgren, 2009 [4]	WHR	G	62	262	303	.31	0.6
LYPLAL1	rs4846567	Heid, 2010 [3]	WHR	G	56	267	312	.30	0.9
LY86	rs1294421	Heid, 2010 [3]	WHR	G	96	281	263	.37	0.1
NFE2L3	rs1055144	Heid, 2010 [3]	WHR	T	413	191	23	.19	0.9
NISCH-STAB1	rs6784615	Heid, 2010 [3]	WHR	T	2	66	567	.06	1.0
RSPO3	rs9491696	Heid, 2010 [3]	WHR	G	168	292	164	.50	0.1
TBX15-WARS2	rs984222	Heid, 2010 [3]	WHR	G	111	302	224	.41	0.6
VEGFA	rs6905288	Heid, 2010 [3]	WHR	A	122	325	191	.45	0.4
ZNRF3-KREMEN1	rs4823006	Heid, 2010 [3]	WHR	A	110	315	211	.42	0.7

freq; frequency
BMI; body mass index
WC; waist circumference
WHR; waist-to-hip ratio
MAF; minor allele frequency

+Surrogate (linked) SNPs: rs4923461 (Speliotes - rs10767664), rs10838738 (Speliotes - rs3817334), rs2568958 (Speliotes - rs2815752), rs10913469 (Speliotes - rs543874), rs7498665 (Speliotes - rs7359397), rs2867125 (Thorleifsson - rs7561317), rs9939609 (Speliotes - rs1558902; Li - rs1121980), rs12970134 (Speliotes - rs571312), rs10146997 (Speliotes - rs10150332)

*With respect to the obesity trait risk-allele. NC/HEZ/HOZ; noncarriers/heterozygotes/homozygotes
†Fisher's exact test.
‡Numbers in DiOGenes for the five last columns were: 435, 188, 21, 0.18, and 0.9.
