# Supplementary material for: TFAP2B Influences the Effect of Dietary Fat on Weight Loss under Energy Restriction
Source: PLoS One. 2012 Aug 27;7(8):e43212. doi: 10.1371/journal.pone.0043212 (PMC3428346; doi:10.1371/journal.pone.0043212)
Supplement: Table S2 — Genes with interesting findings ( P <0.01). Gene and gene-diet interaction effects with P<0.01 in relation to change in weight or waist. (PDF) [file pone.0043212.s004.pdf]

Table S2. Gene and gene-diet interaction effects with *P*<0.01* in relation to change in weight or waist

Gene†	Effect	Effect size reading	Effect size, beta (95% confidence interval)		
			Non-carrier	Heterozygote	Homozygote‡
VEGFA	Gene effect on weight change, *P*=0.001	Weight change, kg, by genotype using non-carriers as reference	*n*=122<br>0 (reference)	*n*=325<br>0.5 (-0.2; 1.1)	*n*=191<br>1.2 (0.4; 1.9)
CTNNBL1	Gene×fat group effect on waist change, *P*=0.0003	Waist change, cm, for high-fat versus low-fat diet in groups of genotype	*n*=543<br>0.8 (0.1; 1.5)	*n*=75<br>-2.7 (-4.5; -0.9)	*n*=3<br>Non-applicable
NPC1	Gene×fat group effect on waist change, *P*=0.008	Waist change, cm, for high-fat versus low-fat diet in groups of genotype	*n*=87<br>2.1 (0.7; 3.5)	*n*=284<br>0.8 (0.1; 1.5)	*n*=240<br>-0.6 (-1.6; 0.5)

*Results for *TFAP2B*×fat group effects are presented separately in Figure 2-3.
†SNP information in Table S1.
‡Homozygous for the obesity trait risk-allele.
