# Supplementary material for: TFAP2B Influences the Effect of Dietary Fat on Weight Loss under Energy Restriction
Source: PLoS One. 2012 Aug 27;7(8):e43212. doi: 10.1371/journal.pone.0043212 (PMC3428346; doi:10.1371/journal.pone.0043212)
Supplement: Table S3 — Sensitivity analysis for weight loss over TFAP2B rs987237 genotypes in NUGENOB. P-value for interaction between TFAP2B and fat group, and beta (95% confidence interval) for fat group by TFAP2B variant, in relation to weight loss, in analyses varying in fat group definition and adjustments, in NUGENOB. (PDF) [file pone.0043212.s005.pdf]

Table S3. *P*-value for interaction between *TFAP2B* and fat group, and beta (95% confidence interval) for fat group by *TFAP2B* variant*, in relation to weight loss, in analyses varying in fat group definition and adjustments, in NUGENOB.

**Weight loss effect**+	**A.** **Original analyses of randomized fat group (*n*=642)**	**B.** **Same as A but only including participants as in case C (*n*=580)**	**C.** **Same as A but including adjust-ment for baseline fat% intake and change in energy intake (*n*=580)**	**D.** **Same as A but only including participants as in case E (*n*=519)**	**E.** **Analyses of reported fat% intake during the intervention of >35 versus <30 (*n*=519)**
P-value	0.00007	0.0001	0.00007	0.005	0.008
Beta: AA	0.99 (0.40; 1.58)	1.15 (0.53; 1.77)	1.08 (0.46; 1.69)	1.06 (0.41; 1.71)	0.99 (0.37; 1.61)
Beta: AG	-0.80(-1.54;-0.06)	-0.67 (-1.43; 0.10)	-0.78 (-1.54; -0.02)	-0.35 (-1.18; 0.48)	-0.15 (-0.95; 0.66)

*Results are not reported for the rare GG variant because results were not applicable in this group after further sample size reduction in analyses B-E.

+P-value for interaction between *TFAP2B* and fat group; beta; estimates for comparison between the high-fat versus the low-fat group.
