# Supplementary material for: TFAP2B Influences the Effect of Dietary Fat on Weight Loss under Energy Restriction
Source: PLoS One. 2012 Aug 27;7(8):e43212. doi: 10.1371/journal.pone.0043212 (PMC3428346; doi:10.1371/journal.pone.0043212)
Supplement: Table S4 — Sensitivity analysis for weight loss over TFAP2B rs987237 genotypes in DiOGenes. P-value for interaction between TFAP2B and fat group, and beta (95% confidence interval) for fat group by TFAP2B variant, in relation to weight loss, in analyses varying in fat group definition and adjustments, in DiOGenes. (PDF) [file pone.0043212.s006.pdf]

Table S4. *P*-value for interaction between *TFAP2B* and fat group, and beta (95% confidence interval) for fat group by TFAP2B variant*, in relation to weight loss, in analyses varying in fat group definition and adjustments, in DiOGenes.

**Weight loss effect⁺**	**A.** **Original analyses of fat% change group, using the median cut-off (*n*=590)**	**B.** **Same as in case A but also including adjustment for change in energy intake (*n*=590)**	**C.** **Analyses of tertile 3 versus 1 of change in fat% intake (*n*=288)**
P-value	0.4	0.4	0.2
Beta: AA	0.09 (-0.50; 0.68)	0.07 (-0.52; 0.66)	0.44 (-0.44; 1.31)
Beta: AG	-0.29 (-1.04; 0.46)	-0.31 (-1.07; 0.44)	-0.37 (-1.44; 0.70)

*Results are not reported for the rare GG variant because results were not applicable in this group, particularly after further sample size reduction in analysis C.

[+] P-value for interaction between *TFAP2B* and fat group; beta; estimates for comparison between the low versus high fat% decrease group.
