# Supplementary material for: TFAP2B Influences the Effect of Dietary Fat on Weight Loss under Energy Restriction
Source: PLoS One. 2012 Aug 27;7(8):e43212. doi: 10.1371/journal.pone.0043212 (PMC3428346; doi:10.1371/journal.pone.0043212)
Supplement: Text S1 — Outline of sensitivity analysis for weight loss over TFAP2B rs987237 genotypes. Includes descriptions of both additional sensitivity analyses for the TFAP2B-dietary fat interaction (results in Table S3 and S4) as well as of analyses based on the general genetic model (SNP coded as a categorical variable, results displayed in Figure S1 and S2). (PDF) [file pone.0043212.s007.pdf]

Text S1. Additional analyses for *TFAP2B*-dietary fat interaction

Sensitivity analyses for additive *TFAP2B*-dietary fat effects

In analyses in NUGENOB of additive *TFAP2B*-fat group interaction in relation to weight loss, we checked whether adjustment for baseline fat% intake and change in energy intake influenced the results (**Table S3**). These results were compared with *TFAP2B*-fat group analyses including participants only for whom dietary intake data were available. Interaction analyses of fat group were also performed for groups of self-reported fat% intake during the intervention lower than 30% versus higher than 35%, in place of the randomized fat groups (**Table S3**).

Similarly, in DiOGenes we checked whether adjustment for change in energy intake influenced the results of additive *TFAP2B*-fat group interaction effects on weight loss (**Table S4**). We also investigated the *TFAP2B*-fat group effect while using more pronounced groups of fat% change; tertile three versus one (**Table S4**).

In essence, these analyses showed no large influence of adjustments for change in energy intake, and in NUGENOB of baseline fat% intake, on *TFAP2B*-dietary fat interactions. Analyses in NUGENOB of fat groups using reported fat% intake during the trial showed somewhat less pronounced gene-fat group effects on weight loss than the use of randomized groups, and analyses in DiOGenes showed somewhat greater gene-fat group effects by the use of more extreme groups for change in fat% intake.

General genetic models for analysis of *TFAP2B*-dietary fat effects

In addition to additive genetic effects investigated throughout the study, for *TFAP2B* we also investigated general genetic effects in interaction with dietary fat, on weight loss and waist reduction. These models were identical to the additive effects analyses, except that the three-level (genotype) SNP-variable of *TFAP2B* was treated as a nominal categorical variable rather than a continuous counterpart, which allows also for estimated non-linear associations. In contrast to the additive model that enforces an additive pattern, the general model shows results for observed data that, however, particularly for small groups may be more strongly influenced by chance.

Compared to the additive model, the general genetic model for *TFAP2B*-dietary fat interaction showed more pronounced effects in relation to weight loss and waist reduction in NUGENOB (**Figure S1-S2**). In DiOGenes, the rare GG variant included only 18 participants in analyses of weight loss and 15 participants in analyses of waist reduction, so analyses of this group was considered too small to provide any meaningful information. In the AA genotype group, beta for weight change in the groups of less than versus more than median fat% reduction was 0.1 (95% CI, -0.5; 0.7), and it was -0.6 (95% CI, -1.4; 0.3) in the AG group. The corresponding for waist change were 0.1 (95% CI, -0.8; 1.0) and -1.4 (95% CI, -2.8; -0.1), respectively.
